# Examinations of the Illinois State Board of Health

**Published:** 1880-06

**Authors:** 


### Domestic Correspondence.

### ARTICLE IX.

### EXAMINATIONS OF THE ILLINOIS STATE BOARD OF HEALTH.

*To the Editors—Gentlemen:* Permit me, through your columns, to ask the members of the Illinois State Board of Health what reasons they have for licensing under-graduates who are still attending medical colleges, and who have not yet completed the ordinary course of reading (three years), to say nothing of lectures?

Why is it that in several instances such students have been licensed, who have never practiced?

And how can we account for the singular coincidence that the students so licensed have, as a rule, been far below the average under-graduate in their class examinations?

The above questions are suggested by four or five cases which have fallen under my personal observation, and with which I am thoroughly conversant.

The same inquiries have doubtless suggested themselves to others, and for them and the profession of the State, as well as myself, I hope the State Board may give a satisfactory explanation.

PRACTITIONER.

[The facts given by our correspondent, are those which are known to members of the profession in this city, and, we have reason to believe, are known also to the members of the board. The trouble seems to be that, given a certain standard, those who are able to meet it, are entitled to certificates. The standard of the board in its examination should be evidently raised above the grade of a first year's medical student, where it is plainly at present.—ED.]

